# Identifying Great Auricular Nerve via Bony Landmarks: A Cadaver Study

**DOI:** 10.1007/s12663-025-02568-3

**Published:** 2025-05-23

**Authors:** May Lin, Faisal Quereshy, Dale Baur

**Affiliations:** Department of Oral and Maxillofacial Surgery, School of Dental Medicine, Case Western Reserve University, 9601 Chester Ave, Cleveland, OH 44106 USA

**Keywords:** Great Auricular Nerve, Cadaver, Head and Neck Anatomy

## Abstract

**Background:**

The great auricular nerve (GAN) is a sensory nerve supplied by C2–C3 that provides sensation to the angle of the mandible and posterior auricle. It is commonly sacrificed in both elective and nonelective surgeries but does not lead to a significant decrease in quality of life.

**Purpose:**

This cadaver study aims to assess two bony landmarks, the angle of the mandible (AOM) and the tip of the mastoid process, and calculate a ratio that can reliably serve as a dependable range for identifying the Great Auricular nerve (GAN) during surgical procedures.

**Study Design:**

Forty-six cadaver sides from the Case Western Reserve University Anatomy Department were dissected to expose the GAN along the AOM, Erb’s point, and mastoid process. This study used two independent observers to calculate the ratio between AOM and mastoid process (mm) divided by the AOM to GAN (mm) along the same angle.

**Analyses:**

Statistical analyses assessed the reliability of the ratio for identifying the GAN. Mean, standard deviation, and coefficient of variation were calculated, and data normality was confirmed. Interobserver reliability was evaluated using the coefficient of variation.

**Results:**

The mean ratio was 0.457 with a standard deviation of ± 0.085. The data was normally distributed with a coefficient of variation was 14.21 for the distance between AOM and GAN and 22.00 for the distance between AOM and the mastoid process.

**Conclusion:**

GAN was reliably found by bisecting about halfway between AOM and mastoid process.

**Supplementary Information:**

The online version contains supplementary material available at 10.1007/s12663-025-02568-3.

## Background and Significance

The Great Auricular Nerve (GAN) is the largest sensory branch of the cervical plexus, and is specifically supplied by C2–3. The GAN will branch off the superficial cervical plexus around Erb’s Point and run anteriorly along the sternocleidomastoid (SCM) [1]. The GAN is divided into two branches: the anterior branch and the posterior branch. The anterior (facial) branch provides cutaneous innervation to the parotid gland, and the posterior (mastoid) branch provides cutaneous innervation to the angle of the mandible and posterior surface of the auricle [2].

GAN sacrifice is common in malignant tumor excisions and radical neck dissections. However, preservation is the standard of care in elective procedures like rhytidectomy, platysmaplasty, and the management of benign pathology. GAN complications like paresthesia, anesthesia, or painful neuromas occur 6–7% [3]. Anatomical variation in length and course of the GAN exists as described by Yang et. al where they characterized five branching types [4]. Therefore, during surgical procedures, GAN can be at risk for damage due to anatomical variations and result in sensory deficits. For essential procedures, such as parotidectomies, intraoperative GAN sacrifice is reported, but there is conflicting evidence if GAN preservation can improve postoperative cutaneous sensation [5]. In addition, there will most likely be sensory deficits, regardless of the preservation or sacrifice of GAN. Loss of GAN function does not significantly impact the quality of life [5–7]. However, efforts should be made to preserve the GAN if possible, especially in elective procedures. For example, in a rhytidectomy, the GAN is the most likely nerve to be injured, with a complication rate of 6–7% annually [8]. Although GAN injury may be unavoidable in certain cases, inflicting iatrogenic nerve injury in elective cosmetic surgeries falls below the standard of care. A careful understanding of the location and course of the GAN during high risk surgical procedures is essential.

Commonly measured bony landmarks include Erb’s Point, McKinney’s Point, nerve point, and punctum nervosum. Erb’s Point is most commonly used to describe the exit of the cutaneous branches of the cervical plexus, located at the posterior border of SCM [9]. However, Raikos et. al argues all four points are misnomers and identifies the GAN via the Great Auricular Point where the nerves exits at the posterior border of SCM [10]. Aside from named points, there are various landmarks used to identify the GAN, and most studies incorporate both soft tissue and bony anatomical landmarks. Several resources investigated the course of GAN along SCM, where the GAN is reliably found within the middle third of the muscle [3, 10, 11]. Additionally, the external jugular vein is reliably found anterior to the GAN [3, 8]. Rohrich et al focused on the preauricular adipose compartments where the main branch of GAN was reproducibly found in the subauricular membrane [12]. While multiple soft tissue landmarks have been identified, hard tissue landmarks are arguably more reliable due to their fixed positioning and resistance to specimen weight. In terms of bony landmarks, commonly measured points include the mastoid process, angle of the mandible, and external auditory canal [3, 11]. Although these reference points exist, there is not a clear consensus on their reliability. The advantages of fixed bony landmarks include their simple identification and consistent measurements intraoperatively, which makes them advantageous over soft tissue anatomical landmarks. Therefore, a reliable anatomical landmark is advantageous; fixed bony landmarks could potentially provide the surgeon with intraoperative guidance to avoid injury. Specifically, the measurement will focus on the ratio at which the AOM and the tip of the mastoid process bisect GAN.

## Methods

**Fig. 1:**
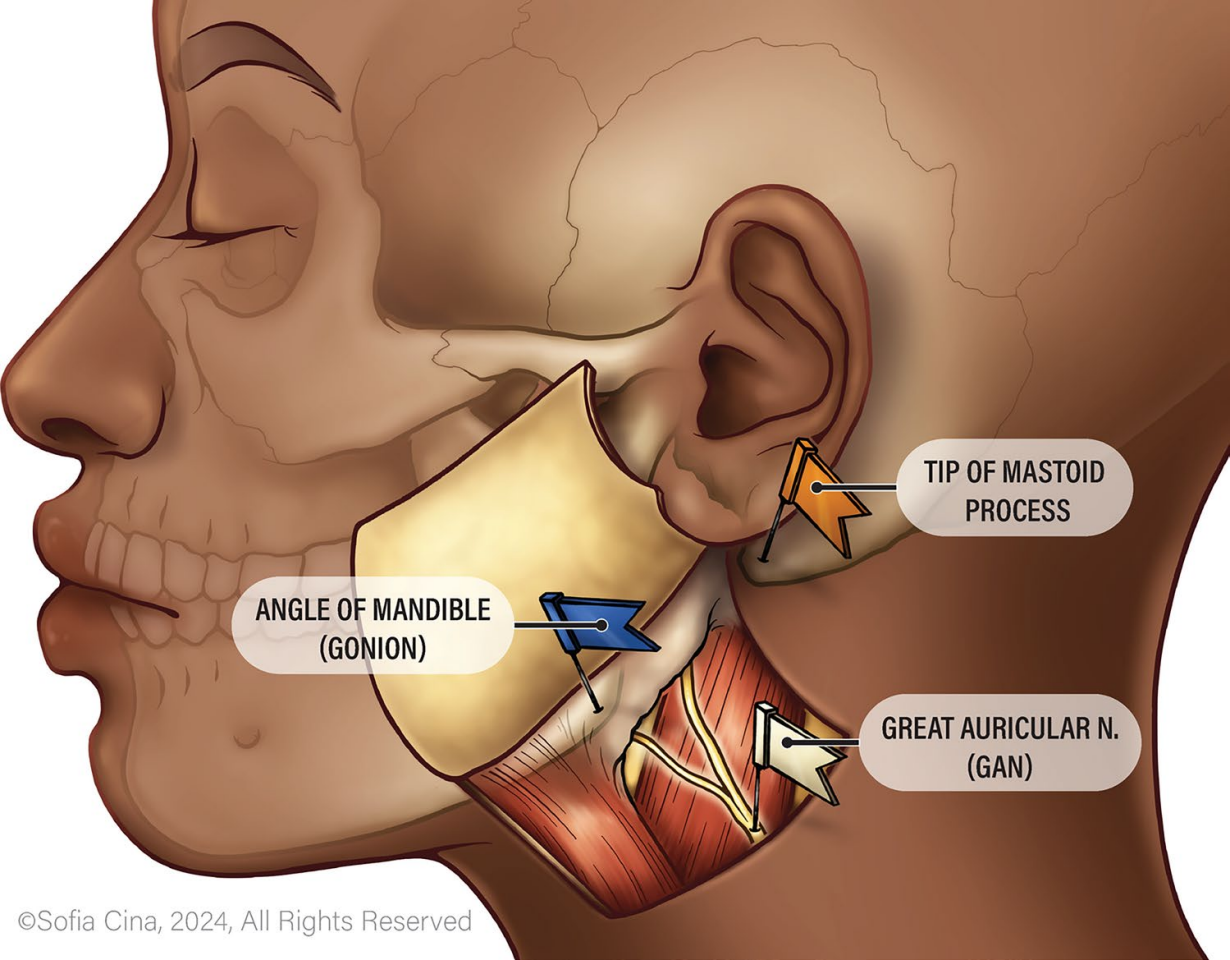
Diagram showing completed cadaver dissection exposing the GAN nerve trunk, with pins identifying the AOM and mastoid process

Cadavers originated from the CWRU School of Medicine Anatomical Donation Program. This study analyzed 27 cadavers, from which a total of 46 heminecks were examined. Some cadavers were intact, allowing for bilateral dissection, while others had been previously dissected, limiting the analysis to a single hemineck. Primary incisions were formed between the following points: Erb’s point, AOM, and the mastoid process. The GAN was dissected from Erb’s point superiorly to the auricle in fascial planes. After the identification of the nerve, three points were established with color-coded pins for measurement: AOM, the mastoid process, and the nerve trunk of GAN (Fig. 1).The great auricular nerve (GAN) was dissected from Erb’s Point superiorly to the auricle through fascial planes. Once identified, three reference points were marked using color-coded pins: AOM, MP, and the GAN trunk (Fig. 1). Two independent researchers took measurements using digital calipers. The following distances were recorded: AOM to MP, AOM to GAN (along the same angle as AOM to MP). The ratio was calculated as the distance from AOM to GAN divided by the distance from AOM to the mastoid process (AOM–GAN/AOM–MP).

All distances were measured in millimeters (mm) to account for anatomical differences between cadavers. Statistical analysis included descriptive statistics such as mean and standard deviation, and distribution curves were generated to assess data normality. Inter-investigator reliability was evaluated using the coefficient of variation. Statistical analysis included descriptive statistics, such as mean and standard deviation, as well as distribution curves to examine the normal distribution of the data. Inter-investigator reliability was calculated using a coefficient of variation to ensure the precision of measurements. All calculations were conducted in Microsoft Excel.

This study was conducted using cadaveric specimens and does not involve human subjects as defined by federal regulations. Therefore, it is exempt from Institutional Review Board (IRB) review in accordance with our institution’s policy. All cadaveric specimens were treated respectfully and handled in accordance with institutional policies and the ethical principles outlined in the Declaration of Helsinki.

## Results

The sample pool consisted of 16 females and 11 males, however, some cadavers were previously dissected allowing investigators to measure only one side. Additionally, five of the cadavers were prosected heads rather than full bodies. All cadavers were reported to have two branches, with an anterior branch toward the parotid gland and a posterior branch toward the auricle.

**Fig. 2:**
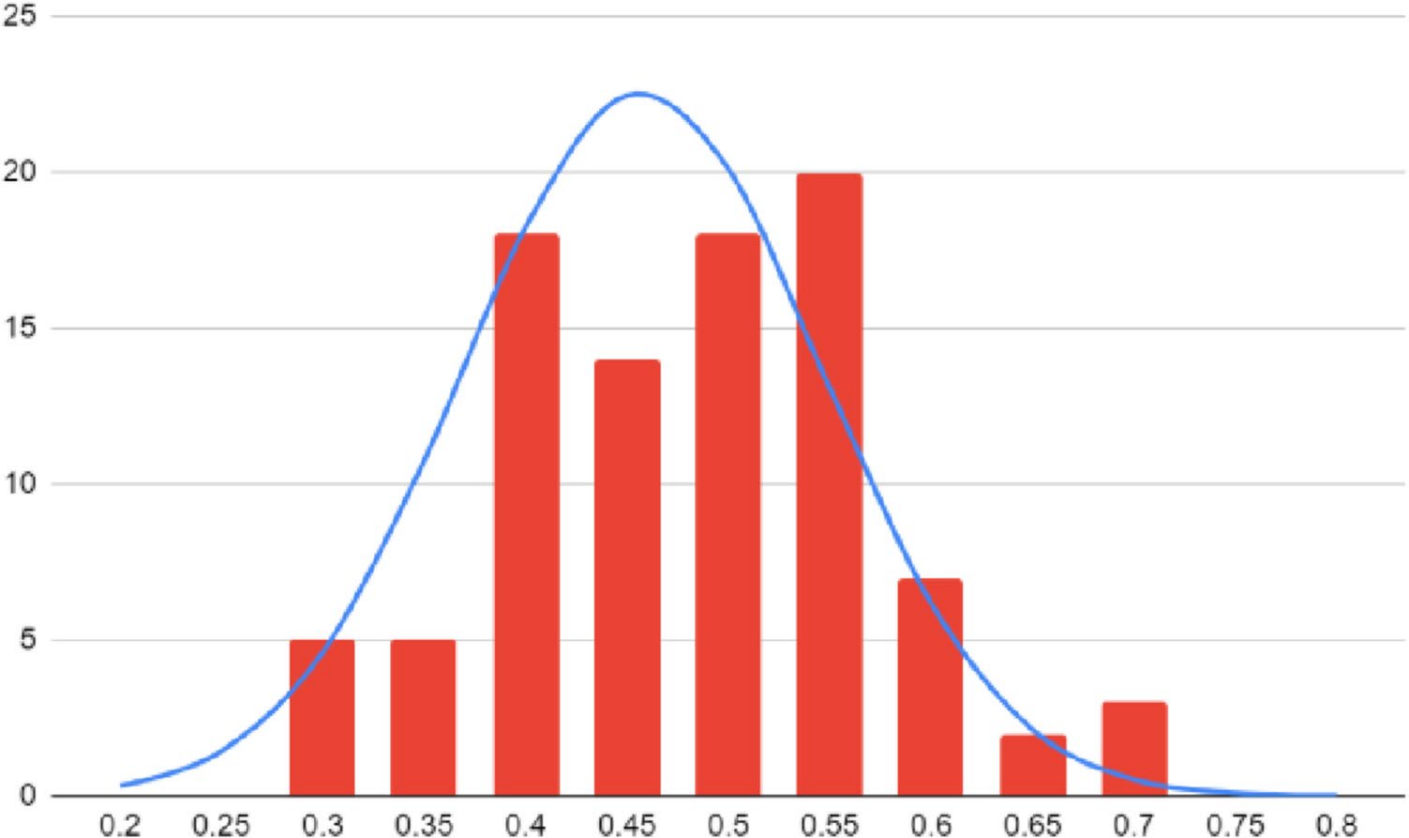
Distribution of Data Under a Normal Curve

Statistical analysis shows the data follows a normal curve with a bimodal distribution (Fig. 2). Between the two observers, the coefficient of variation was 14.21 for the distance between AOM and GAN, and 22.00 for the distance between AOM and the mastoid process.

For descriptive statistics, the mean of the ratios was reported as 0.457 with a standard deviation of ± 0.085. While measurements varied greatly between cadavers, the ratios were standardized across the sample pool. Table 1 includes minimums, maximums, means, and standard deviations for each measurement and calculation.

**Table 1:** Descriptive Statistics of Measurements, including minimum measurement, maximum measurement, mean of measurements, and standard deviations

	Min	Max	Mean	Standard deviation
Distance of AOM to GAN (mm)	19.1	49.0	32.5	7.1
Distance of AOM to MP (mm)	50.6	95.2	71.3	10.1
Ratio of AOM to GAN (AOM–GAN/AOM–MP)	0.285	0.667	0.457	0.085

Measurements were divided into distance of AOM to GAN, distance of AOM to MP, and ratio of AOM to GAN. AOM = Angle of Mandible, GAN = Nerve Trunk of Great Auricular Nerve, MP = Mastoid Process

## Discussion

Most research on the GAN has focused on soft tissue landmarks like Erb’s Point, but this study provides new evidence for using bony landmarks in preventing iatrogenic injury. While previous studies have compared bony landmarks for identifying the GAN, this is the first to establish a reliable ratio between them, offering a reproducible guide that can be used intraoperatively to avoid iatrogenic nerve damage.

There are several limitations of this study including cadaver variability and distortions created by the cadaver preservation process. Furthermore, the study did not assess variations of the contralateral side, as some dissections were performed on heminecks rather than complete bilateral dissections. This limits the generalizability of findings to both sides of the neck.

We also failed to collect data on the specimen’s race, which could influence bony landmarks. Additionally, BMI seems to influence the identification of the nerve, as increased subcutaneous fat seems to displace the nerve closer to AOM, but more research is required for further explanation. However, in the context of cosmetic surgery, these limitations may not pose a complication. In the future, increased sample sizes can provide a more accurate calculated ratio.

## Conclusion

This study reliably concludes the GAN, specifically the nerve trunk, consistently passes near the midpoint between AOM and MP, with a slight anterior shift toward AOM. This ratio offers a reliable anatomical reference for identifying the GAN intraoperatively and reducing the risk of iatrogenic injury. Future research with a larger dataset is necessary to refine the confidence interval of this ratio within the bounds of limitations for this study. This bony landmark is proposed to identify GAN to prevent iatrogenic injury during surgery. We propose further research with a larger sample size and greater inclusion of relevant factors to reduce the confidence interval of this ratio.

## Supplementary Information

Below is the link to the electronic supplementary material.

Supplementary file1 (JPG 311 kb)
