# Supplementary figures and images for: Identifying Great Auricular Nerve via Bony Landmarks: A Cadaver Study
Source: J Maxillofac Oral Surg. 2025 May 23;25(3):814–7. doi: 10.1007/s12663-025-02568-3 (PMC13201816; doi:10.1007/s12663-025-02568-3)

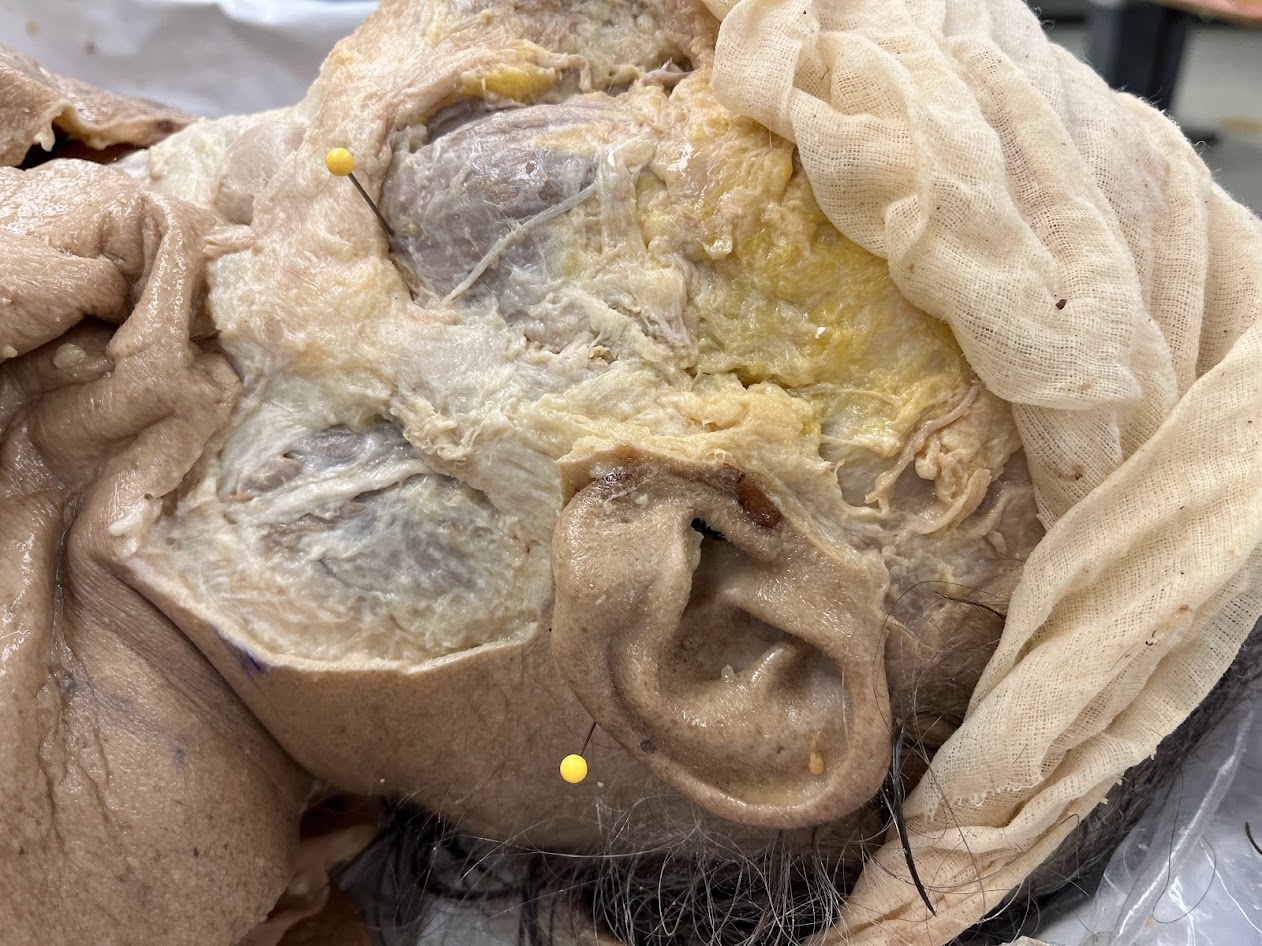

Supplement: Supplementary file 1 — Supplementary file1 (JPG 311 kb) [file 12663_2025_2568_MOESM1_ESM.jpg]
